# Persistent Lactic Acidosis Portending a Poor Prognosis in Extensive Metastatic Signet Ring Cell Carcinoma of the Cecum

**DOI:** 10.7759/cureus.78762

**Published:** 2025-02-09

**Authors:** Miki Yokokawa, Yoshito Nishimura, Sean Saito, Kathy Ho, Christina Chong

**Affiliations:** 1 Internal Medicine, University of Hawaii, Honolulu, USA; 2 Pathology, University of Hawaii, Honolulu, USA; 3 Internal Medicine, The Queen's Medical Center, Honolulu, USA

**Keywords:** lactic acidosis, metastatic cancer, signet ring cell carcinoma of the cecum, tumor burden, warburg effect

## Abstract

Lactic acidosis is an uncommon metabolic complication of malignancy, often associated with high tumor burden and increased mortality, and more frequently observed in hematologic malignancies than in solid tumors. This case report describes a patient with newly diagnosed signet ring cell carcinoma of the cecum, an uncommon and aggressive histological subtype of colon cancer, complicated by severe type B lactic acidosis.

A 66-year-old female patient with primary signet ring cell carcinoma of the cecum and peritoneal carcinomatosis underwent a right colectomy with extended small bowel resection. Two months later, she presented to the emergency department with a partial small bowel obstruction, and laboratory studies revealed a markedly elevated lactic acid level. The patient's lactic acidosis levels remained persistently elevated despite supportive interventions, and she passed away on the fifth day of hospitalization.

Cancer cells may overproduce lactate through aerobic glycolysis, known as the Warburg effect. Although rare in solid tumors, one should have a high suspicion for type B lactic acidosis in oncology patients given the associated poor prognosis and high mortality.

## Introduction

Type B lactic acidosis is an uncommon complication of aggressive tumors and portends a poor prognosis [1,2]. Tumor cells consume large amounts of glucose, producing substantial lactate even in the presence of oxygen [3]. Acidification of the tumor microenvironment drives metastasis, promotes angiogenesis, and induces immunosuppression, all linked with poorer clinical outcomes. The majority of type B lactic acidosis cases have been associated with hematologic malignancies (87%), while those related to solid tumors were less common, accounting for only 13% [4,5]. We report a case of primary signet ring cell carcinoma of the cecum accompanied by peritoneal carcinomatosis and persistently elevated lactic acid levels.

## Case presentation

A 66-year-old female patient with a medical history significant for ductal carcinoma in situ of the left breast status post lumpectomy, right renal cell carcinoma status post cryoablation, non-insulin-dependent diabetes mellitus, and a recent diagnosis of signet ring cell carcinoma of the cecum with peritoneal carcinomatosis presented to the emergency department with nausea, vomiting, and difficulty tolerating oral intake over the past few days. She denied fever, chills, abdominal pain, hematemesis, melena, or hematochezia.

**Figure 1:**
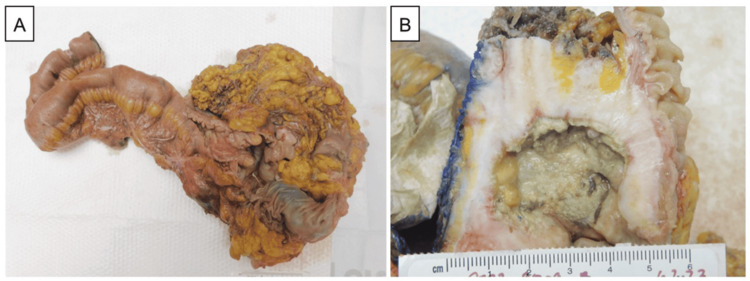
Gross specimen An image shows the right ileocolectomy including the terminal ileum, right colon, and appendix (A). A photograph highlights diffuse wall thickening of the cecum by tumor with extension into the subserosal fat (B).

**Figure 2:**
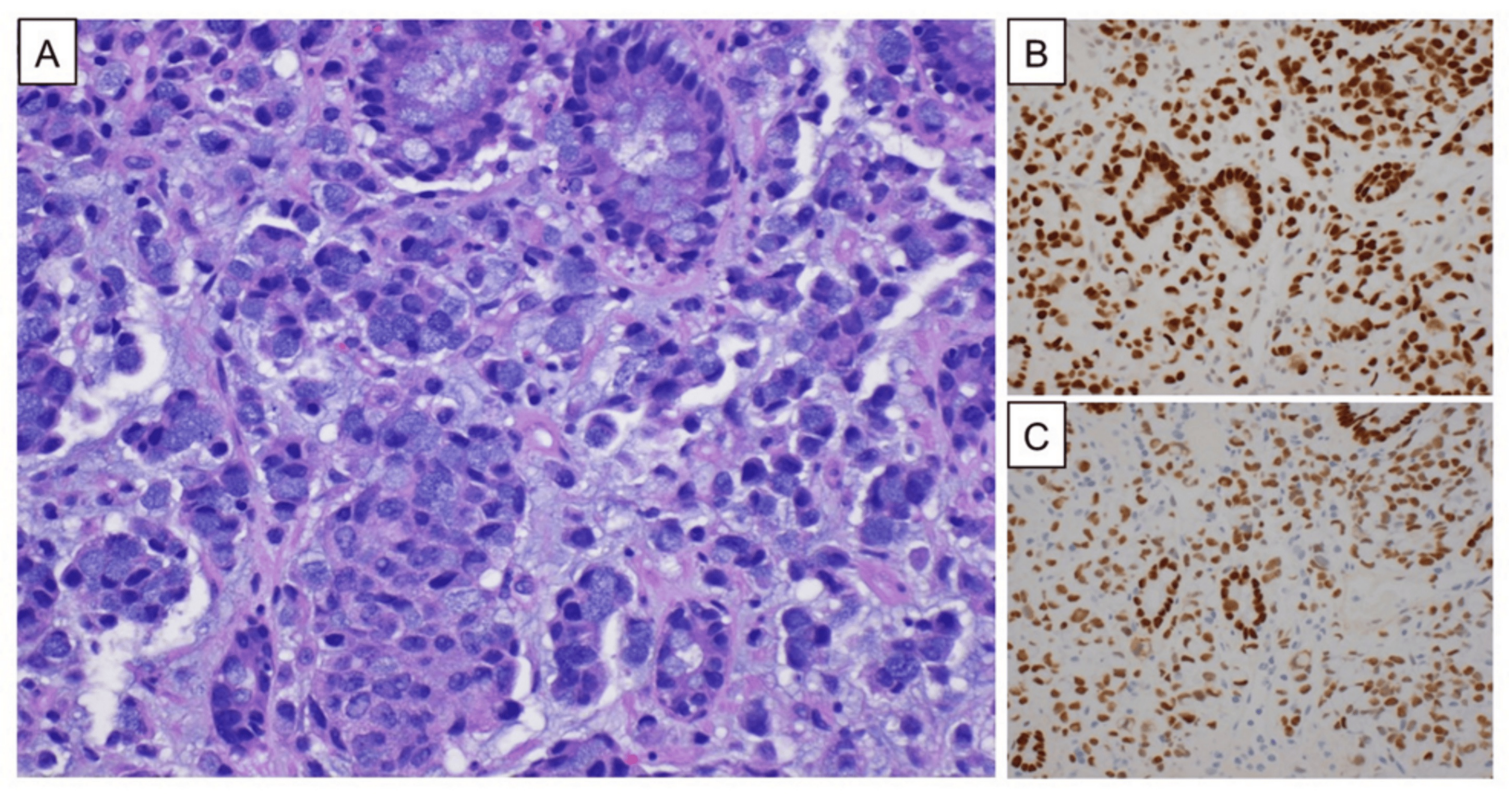
HE and IHC staining of the biopsy specimens of cecal tumors A microphotograph shows the proliferation of tumor cells. The tumor cells contain abundant mucin, pushing the nucleus to the periphery, demonstrating a "signet ring" cell morphology (HE ×400, A). The tumor cells show the immunohistochemical expression of CDX-2 (B) and SATB2 (C), which is suggestive of colorectal origin (IHC ×400). HE: hematoxylin-eosin; IHC: immunohistochemical

**Figure 3:**
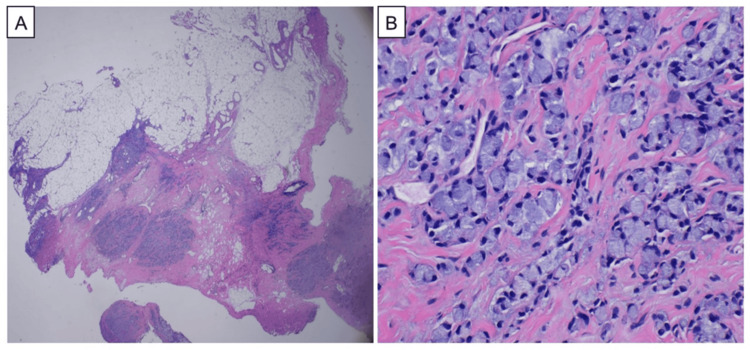
HE staining of the biopsy specimens of the omentum A microphotograph reveals the proliferation of tumor cells infiltrating the omental tissue (HE ×20, A). The tumor cells contain abundant mucin, pushing the nucleus to the periphery, demonstrating a "signet ring" cell morphology (HE ×400). HE: hematoxylin-eosin

Two months prior to presentation, the patient underwent a surveillance computed tomography (CT) of the abdomen for her renal cell carcinoma, which revealed cecal thickening concerning for malignancy. A subsequent colonoscopy identified an ulcerated cecal mass, and pathology confirmed poorly differentiated adenocarcinoma with signet ring cells. Her carcinoembryonic antigen level was elevated at 36.3 ng/mL. Upon inspecting the abdomen during surgery, diffuse peritoneal nodules were observed in the lower anterior abdominal wall near the pelvis, along the falciform ligament, and throughout the mesentery. The cecum was found to be invading the abdominal wall, with two small bowel implants causing intussusception. Additionally, there was extensive carcinomatosis throughout the small bowel mesentery, along with a large, hemorrhagic cecal mass infiltrating the abdominal wall. No focal lesions were noted on the liver. Due to concerns for future complications of obstruction and bleeding, she underwent a palliative right colectomy with an extended small bowel and lateral abdominal wall muscle resections (Figure 1A, 1B). Surgical pathology revealed pT4 N2b M1c grade 3, poorly differentiated adenocarcinoma with signet ring cells (Figure 2A), invading the visceral peritoneum (Figure 3A, 3B). Multiple additional tumor nodules were identified in the omentum and ileum. Immunohistochemistry stains for CDX-2 and SATB2 were positive in tumor cells (Figure 2B, 2C), consistent with a colorectal origin. There was no immunohistochemical evidence of mismatch repair deficiency or microsatellite instability. The patient was discharged but did not follow up with oncology for planned adjuvant chemotherapy, citing concerns about potential side effects.

**Figure 4:**
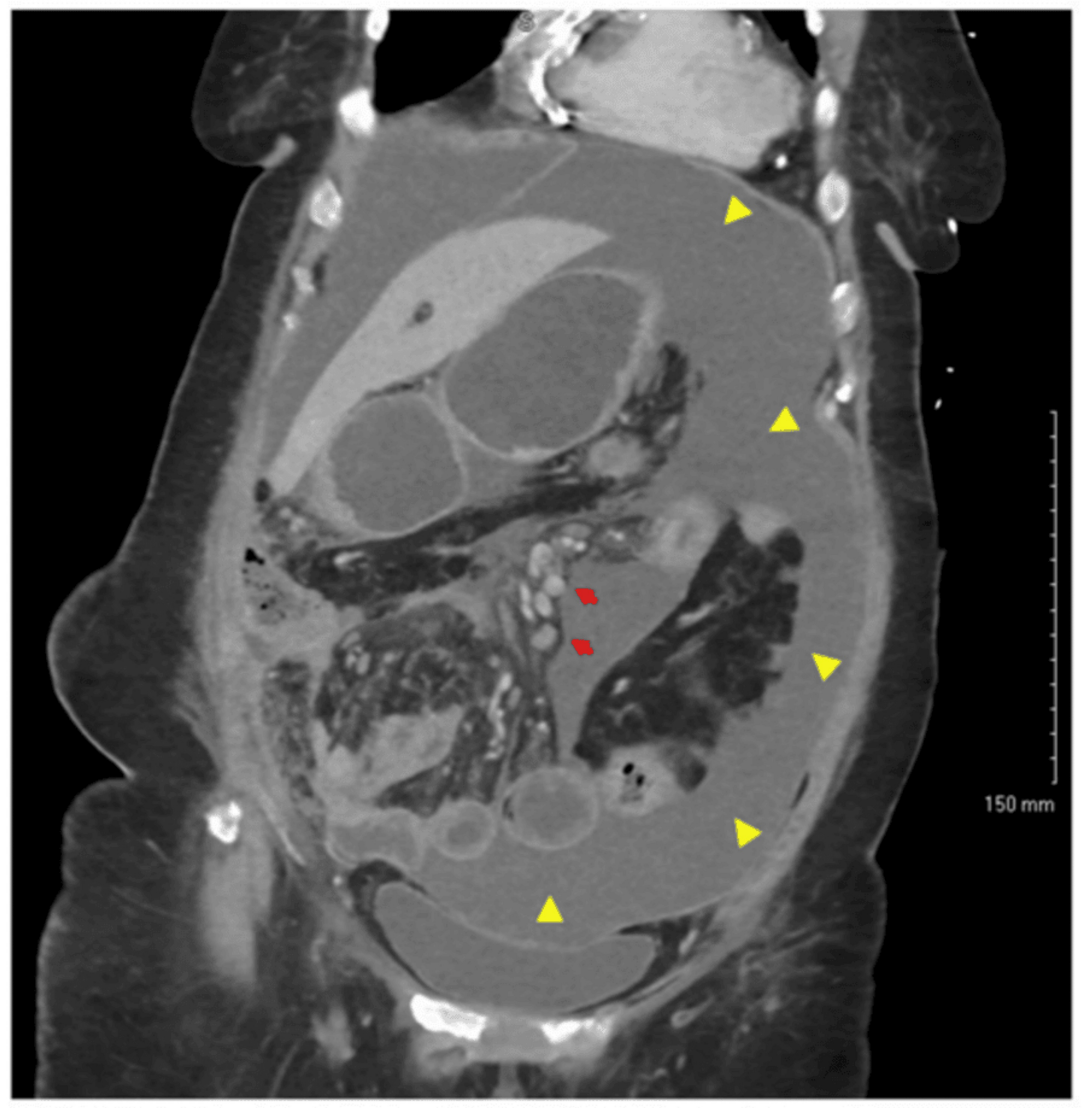
CT of the abdomen and pelvis with contrast Known peritoneal carcinomatosis. Moderate amount of ascites (yellow arrowheads) and retroperitoneal and mesenteric lymphadenopathy (red arrows). Mildly dilated loops of small bowel suspected partial small bowel obstruction. CT: computed tomography

In the emergency department, the patient was afebrile and normotensive but tachycardic with a heart rate of 130 beats per minute. Physical examination revealed abdominal distention with hypoactive bowel sounds and tenderness in the right lower quadrant, accompanied by voluntary guarding but no rebound tenderness. Laboratory studies were significant for high anion gap metabolic acidosis, lactic acidosis, and acute kidney injury. Table 1 summarizes the main laboratory findings on admission. CT scan of the chest, abdomen, and pelvis revealed findings consistent with peritoneal carcinomatosis, including a new moderate amount of ascites, retroperitoneal and mesenteric lymphadenopathy, omental nodularity, and dilated loops of small bowel concerning for partial small bowel obstruction (Figure 4). Initial treatment included fluid resuscitation and empiric antibiotics. A nasogastric tube was placed on low intermittent suction. An abdominal X-ray with contrast revealed contrast in the descending colon and cecum. Intermittent bowel obstruction was suspected, likely due to a high tumor burden and adhesions. Tube feeding was initiated. Blood cultures were negative.

**Table 1:** Initial laboratory data

	Result	Reference range
Complete blood count		
White blood count (×10^3^/uL)	10.76	3.80-10.80
Hemoglobin (g/dL)	8.5	13.7-17.5
Hematocrit (%)	26.5	40.1-51.0
Platelet count (×10^3^/uL)	232	151-424
Biochemical findings		
Glucose (mg/dL)	177	70-99
Blood urea nitrogen (mg/dL)	33	6-23
Creatinine (mg/dL)	1.5	0.6-1.4
Sodium (mEq/L)	132	133-145
Potassium (mEq/L)	3.6	3.3-5.1
Chloride (mEq/L)	91	95-108
Anion gap (mEq/L)	24	14-20
Phosphorus (mg/dL)	4.0	2.5-4.5
Magnesium (mg/dL)	1.8	1.6-2.6
Lipase (U/L)	133	13-60
Aspartate aminotransferase (IU/L)	40	0-40
Alanine aminotransferase (IU/L)	8	0-41
Alkaline phosphatase (IU/L)	96	35-129
Total bilirubin (mg/dL)	1.2	0-1.2
Total protein (mg/dL)	7.7	6.4-8.3
Albumin (mg/dL)	3.3	3.5-5.2
Lactic acid (mEq/L)	7.5	0.5-2.2
Other pertinent findings		
Serum osmolality (mOsm/kg)	296	280-300
Beta-hydroxybutyrate (mmol/L)	2.74	0.02-0.27
Venous blood gas		
pH	7.38	7.38-7.42
pCO2 (mmHg)	29	38-42
HCO3 (mmol/L)	17	22-26

**Figure 5:**
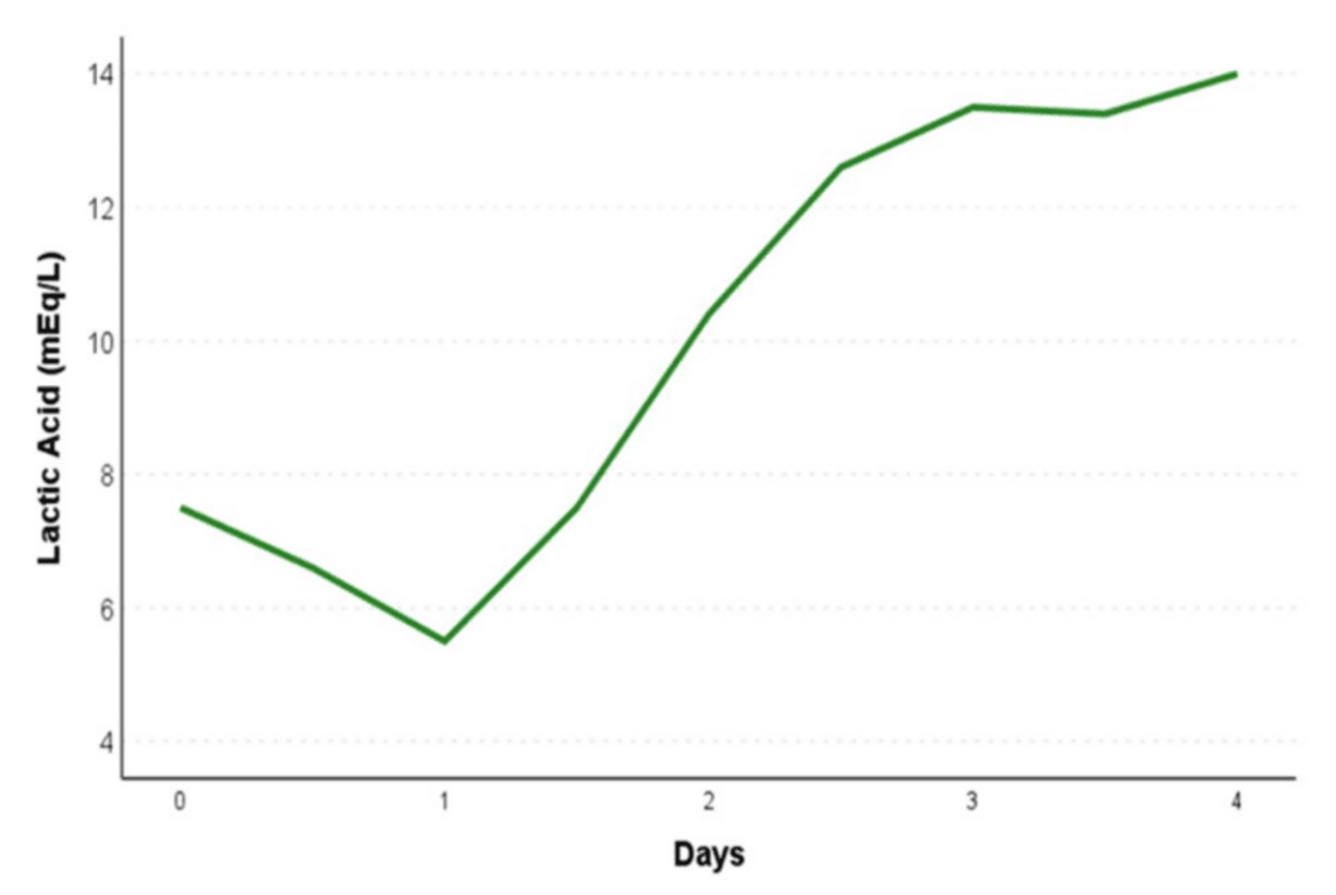
Trend of serum lactic acid levels The line graph depicts the serum lactic acid levels after the hospital admission.

The patient was also treated for possible euglycemic diabetic ketoacidosis with fluid replacement and insulin therapy. Despite these interventions and the absence of systemic hypoperfusion, including hypotension, sepsis, oligoanuria, or impaired mental status, the patient continued to experience refractory, worsening high anion gap metabolic acidosis with a subsequent pH drop to 7.08 and rising lactic acid levels exceeding 14 mEq/L by day 4 (Figure 5, Table 2). The acidosis was therefore attributed to type B lactic acidosis related to her malignancy. Given that the rapid clinical decline was mainly driven by malignancy, inpatient palliative chemotherapy was offered. However, the patient declined the cancer-directed therapy and passed away on day 5.

**Table 2:** Main laboratory data trends

Hospital day	0	1	2	3	4
pH	7.38	7.45	-	-	7.08
HCO3 (mmol/L)	17	17	-	-	9
Anion gap (mEq/L)	24	21	27	29	38
Lactic acid (mEq/L)	7.5	5.5	12.6	13.5	>14
Creatinine (mg/dL)	1.5	0.9	0.9	0.8	1.0

## Discussion

Lactic acidosis is a common cause of metabolic acidosis in hospitalized patients and is generally defined as a serum lactate concentration greater than 4 mmol/L [6]. The causes of lactic acidosis are classified into three types: type A, type B, and type D. Type A lactic acidosis is typically associated with marked tissue hypoperfusion, often due to conditions such as shock or severe hypoxemia. Type D lactic acidosis is a rare form of lactic acidosis, typically observed in patients with short bowel syndrome or gastrointestinal malabsorption. In contrast, type B lactic acidosis occurs in the absence of overt systemic hypoperfusion. It is linked to underlying conditions such as impaired liver function, diabetes mellitus, malignancies, alcoholism, and drug-induced mitochondrial dysfunction [7]. Although type B lactic acidosis is associated with metformin use, the patient was not taking metformin. The patient had no history of alcohol consumption. 

This case illustrates severe, progressive lactic acidosis in a patient with a metastatic solid tumor. Type B lactic acidosis in cancer patients is rare but carries a high mortality rate, often reflecting advanced disease and significant tumor burden. The pathogenesis of type B lactic acidosis remains unclear, but the Warburg effect has been described as a likely hypothesis where tumor cells preferentially produce excessive lactate through aerobic glycolysis despite the presence of adequate oxygenation [8]. Lactate is critical in driving key processes throughout the major stages of carcinogenesis. Reduced hepatic clearance, frequently observed in the presence of liver metastases, is another proposed mechanism in the pathogenesis of type B lactic acidosis. Liver involvement has been reported in over 90% of solid tumor cases associated with type B lactic acidosis [7]. Our patient, however, showed no signs of liver metastases on imaging.

The prognosis of type B lactic acidosis depends on the underlying cause, the speed of correction, and the lactate levels. Mortality increases proportionally with the duration and severity of lactic acidosis. Despite aggressive management, type B lactic acidosis is associated with a poor prognosis, with reported mortality rates exceeding 90% [4]. Early identification of the underlying cause of lactic acidosis is crucial for guiding management and determining prognosis. Type A and B lactic acidosis often overlap, making the differentiation a challenge. Initial treatment focuses on supportive care and addressing possible underlying conditions, such as sepsis or other causes of hypoperfusion. In the present case, the temporary improvement in lactic acid levels following fluid replacement on the day after hospital admission suggests that the patient likely experienced a mixed picture of type B lactic acidosis superimposed on type A pathophysiology driven by hypovolemia secondary to inadequate oral intake in the setting of her nausea and vomiting. Other possible supportive management strategies for type B lactic acidosis, including bicarbonate infusions, thiamine administration, and renal replacement therapy, have not consistently shown benefit [8].

Cancer-directed therapy is the primary treatment for type B lactic acidosis caused by an underlying malignancy, particularly in cases with a high tumor burden [9]. However, remission of acidosis is reported in only 10% of patients, and the overall prognosis remains poor [10]. Our patient was diagnosed with signet ring cell carcinoma of the cecum, an aggressive histological subtype of colon cancer, accounting for less than 1% of all colon cancers. This subtype is characterized by high aerobic glycolytic activity [11]. Clinical symptoms of signet ring cell carcinoma typically appear late in its progression, leading to most cases being diagnosed at an advanced stage. Consequently, the overall survival rate is significantly lower than conventional colorectal adenocarcinoma [12]. Another reported case described signet ring cell carcinoma complicated by lactic acidosis; however, the lactic acidosis was attributed to type A lactic acidosis secondary to sepsis in the setting of ulcerative colitis [13]. 

## Conclusions

Type B lactic acidosis is an uncommon complication of aggressive tumors. Overproduction of lactate may occur in the cancer cells themselves by aerobic glycolysis, a phenomenon known as the Warburg effect. Refractory type B lactic acidosis in patients with solid tumors may reflect a significant disease burden with a high likelihood of rapid progression. This condition requires comprehensive clinical evaluation to exclude alternative causes and, when aligned with the patient's goals of care and prognosis, the timely initiation of cancer-directed therapy.
